# Sixty Hertz Neurostimulation Amplifies Subthalamic Neural Synchrony in Parkinson’s Disease

**DOI:** 10.1371/journal.pone.0121067

**Published:** 2015-03-25

**Authors:** Zack Blumenfeld, Anca Velisar, Mandy Miller Koop, Bruce C. Hill, Lauren A. Shreve, Emma J. Quinn, Camilla Kilbane, Hong Yu, Jaimie M. Henderson, Helen Brontë-Stewart

**Affiliations:** 1 Department of Neurology and Neurological Sciences, Stanford University, Stanford, California, United States of America; 2 Department of Neurosurgery, Stanford University, Stanford, California, United States of America; University of Toronto, CANADA

## Abstract

High frequency subthalamic nucleus (STN) deep brain stimulation (DBS) improves the cardinal motor signs of Parkinson’s disease (PD) and attenuates STN alpha/beta band neural synchrony in a voltage-dependent manner. While there is a growing interest in the behavioral effects of lower frequency (60 Hz) DBS, little is known about its effect on STN neural synchrony. Here we demonstrate for the first time that during intra-operative 60 Hz STN DBS, one or more bands of resting state neural synchrony were amplified in the STN in PD. We recorded intra-operative STN resting state local field potentials (LFPs) from twenty-eight STNs in seventeen PD subjects after placement of the DBS lead (model 3389, Medtronic, Inc.) before and during three randomized neurostimulation sets (130 Hz/1.35V, 130 Hz/2V, 60 Hz/2V). During 130 Hz/2V DBS, baseline (no DBS) STN alpha (8 – 12 Hz) and beta (13 – 35 Hz) band power decreased (N=14, P < 0.001 for both), whereas during 60 Hz/2V DBS, alpha band and peak frequency power increased (P = 0.012, P = 0.007, respectively). The effect of 60 Hz/2V DBS opposed that of power-equivalent (130 Hz/1.35V) DBS (alpha: P < 0.001, beta: P = 0.006). These results show that intra-operative 60 Hz STN DBS amplified whereas 130 Hz STN DBS attenuated resting state neural synchrony in PD; the effects were frequency-specific. We demonstrate that neurostimulation may be useful as a tool to selectively modulate resting state resonant bands of neural synchrony and to investigate its influence on motor and non-motor behaviors in PD and other neuropsychiatric diseases.

## Introduction

High frequency subthalamic nucleus (STN) deep brain stimulation (DBS) provides consistent long-term improvement of the cardinal motor signs of Parkinson’s disease (PD) [1–6]. STN DBS and dopaminergic medication attenuate pathological neuronal oscillations and neural synchrony in the alpha/beta band (8–30 Hz) in the Parkinsonian STN, and the degree of attenuation by either therapy has been correlated with the degree of improvement in bradykinesia and rigidity [7–20]. This has led to the suggestion that neural synchrony in the alpha/beta band is related to Parkinsonian limb motor disability, and that successful high frequency DBS overrides pathological neural oscillations and synchrony in the sensorimotor network [21,22].

The mechanism of high frequency DBS is not clearly understood. Experimental and theoretical evidence have suggested a dual effect of cell soma inhibition and axonal excitation [22–26], which may lead to several changes in the local and downstream neural network. Another theory is that high frequency DBS attenuates pathological resting state neural synchrony in the alpha/beta band in the STN region, either by affecting local neuronal oscillations or by suppressing cortical beta band synchrony that was transmitted to the STN via the striatum or directly via the hyperdirect pathway (HDP) [7–9,18,20,27–32]. Until recently, it was difficult to measure the real-time effect of high frequency STN DBS on STN neural synchrony due to the eclipsing of the underlying neural activity by the stimulation artifact. Using an analog filter, we and others have successfully recorded STN local field potentials (LFPs) during high frequency STN DBS and have confirmed that an effect of high frequency STN DBS is to attenuate resting state beta band neural synchrony in a voltage-dependent manner in the STN region [10,18,33–35]. No such investigation has been performed yet on the effect of different frequencies of DBS on STN neural synchrony, even though low (60–80 Hz) DBS may have different effects on motor behaviors than high frequency DBS does [36–41].

In this study, we investigated whether the effect of low frequency STN DBS on resting state neural synchrony in the STN in PD was the same or different from that seen at high frequency and, if different, whether this was due to a different total power delivered or due to frequency itself. We used the lowest frequency of DBS (60 Hz) during which we could reliably distinguish the 8–35 Hz range from the stimulation artifact. This is the first study to determine the effect of intra-operative 60 Hz DBS on neural synchrony in awake human subjects with PD.

## Methods

### Patient assessment

Intra-operative LFP recordings were collected in the operating room immediately after DBS lead (model 3389, Medtronic, Inc., Minneapolis, MN, USA) implantation in twenty-eight subthalamic nuclei from seventeen subjects with PD, off medication. All subjects signed a written consent for the study, and the study as well as the patient consent form were approved by the Stanford Institutional Review Board. The pre-operative selection criteria and assessment of subjects have been previously described [7,28,42]. Long-acting dopaminergic medication was withdrawn over twenty-four hours before and short-acting medication was withdrawn over twelve hours prior to DBS lead implantation. LFP recordings were taken after the therapeutic effectiveness of the DBS lead placement was verified by the absence of adverse effects and an improvement in motor signs [7,43]. Subjects were awake and not on any benzodiazepine medication.

### Intra-operative LFP recordings and experimental protocol

The DBS lead was implanted in the sensorimotor region of the STN using frameless stereotactic technique and multi-pass microelectrode recording (MER) [44]. The base of electrode 0 was placed at the ventral border of the STN, which had been determined during MER. LFPs were recorded differentially from the DBS lead electrodes 0 and 2 before and during constant voltage stimulation was delivered through electrode 1 using an external stimulator (model 3625 screener, Medtronic, Inc., Minneapolis, MN, USA) with the current return through the guide tube dorsal to the electrode [18]. Limb and head movements were monitored using angular velocity sensors on the limbs (Motus Bioengineering, Inc., Benicia, CA, USA), an accelerometer placed on the forehead, surface electromyography over the forearm flexor and extensor muscles, continuous synchronized full-body videography, and intra-operative notes. Patients were instructed to lie still without speaking while keeping their eyes open; the neurologist (HBS, CK) monitored them continuously. After thirty seconds of baseline recording without stimulation, twenty-second epochs of stimulation were applied in randomized order, separated by additional twenty- to thirty-second periods without stimulation. The DBS parameter sets used were: 130 Hz/1.35V, 130 Hz/2V, and 60 Hz/2V. DBS at 130Hz/1.35V had equivalent power to 60 Hz/2V DBS. This was calculated according to the total equivalent energy delivered (TEED) formula: TEED = [voltage^2^ * frequency * pulse width]/impedance [45]. We will refer to this as power, not energy. Stimulation pulse width was sixty microseconds.

### Data acquisition and analysis

LFP signals were pre-amplified with a gain of sixteen by an isolated amplifier (BioAmp 100, Axon Instruments, Inc.) and then passed through a Brownlee Precision 440 Amplifier/Filter (8-pole Bessel low-pass/high-pass filter) followed by an Axon Cyberamp Amplifier/Filter (4-pole Bessel low-pass/high-pass filter), providing a total gain of 50,000. The corner frequencies of the filters were 1 Hz and 30 Hz. The 30 Hz low-pass filter corner frequency was chosen to attenuate the stimulation artifacts produced by 60 Hz stimulation and thereby improve the dynamic range of the digitization process. The kinematic signals (from the accelerometer and angular velocity sensors, all sampled at 1 kHz), the electromyographic signals (sampled at 2 kHz), and the video recording (thirty frames/second) were acquired concurrently with the LFP signals (sampled at 1 kHz) using a data acquisition interface (Power1401) and Spike software (version 2.7) (Cambridge Electronic Design, Ltd., Cambridge, England). Signal analysis was performed in MATLAB (version 8.2, The Mathworks, Inc., Natick, MA, USA). Prior to analysis, the data were parsed into epochs using Spike software for each side corresponding to a baseline state and the various stimulation states, with care being taken to exclude any sections containing signal or movement artifacts from further analysis. Transient voluntary movements and involuntary movements, such as tremor and dyskinesias, may attenuate LFP power and therefore make subsequent power calculations inaccurate [7,32,46–48]. If an inter-stimulation interval was used as the baseline, the power analysis was performed on the interval beginning at least ten seconds after stimulation had ended to avoid including persistent DBS-induced LFP power attenuation [7]. Each epoch contained at least ten seconds of data. Spectrograms of LFP epochs were generated using a short-time Fourier transform of a one second Hanning filtered sliding window and 50% overlap. The power spectral density (PSD) estimate was calculated using Welch’s method with the aforementioned windows and overlap parameters [49]. The frequency response of the filters was measured using a continuous train of rectangular pulses as input signal. Both input and output signals were recorded and the PSD estimate of each was computed. For each 1 Hz frequency component up to 35 Hz, a multiplicative compensation factor was computed to make the output spectrum match the input spectrum. These compensation factors were then applied to our recorded physiological signal PSD estimates to produce a flat frequency response up to 35 Hz.

### Alpha/beta band analysis

LFP power during each stimulation epoch was normalized to the baseline LFP power for an individual hemisphere. Specifically, the LFP power was calculated for two bands of interest: alpha (8–12 Hz) and beta (13–35 Hz). For a given side, the absolute power values in the bands of interest for all epochs were divided by the corresponding absolute power values of the bands of interest from baseline.

### Peak detection and analysis

The frequency resolution of the PSD estimate was 1 Hz. Spectral peaks were detected using a conservative algorithm we have described previously [28]. Peaks were defined as local elevations of spectral power and detected using a 12 Hz sliding window. Within the band of interest (8–35 Hz), the power of the maximum peak during DBS was normalized to the power of the maximum peak at baseline.

### Statistics

Normalized alpha/beta band power was analyzed using a one-way repeated measures ANOVA and Bonferroni correction to determine significant differences in LFP power among the periods of interest across all hemispheres. A paired t-test was used to compare normalized peak power during 60 Hz DBS to baseline across subjects.

## Results

Fourteen out of twenty-eight sides were excluded from all analyses due to the presence of either artifact or movement that corrupted any of the four main epochs of interest (baseline, 130 Hz/2V, 60 Hz/2V, and 130 Hz/1.35V) such that less than ten seconds of usable data for any of them remained. The age (mean ± standard deviation) of the remaining thirteen patients was 64.0 ± 7.9 years at the time of surgery, disease duration from the onset of symptoms was 9.3 ± 5.4 years, and disease duration from the time of diagnosis was 8.0 ± 4.5 years. The UPDRS III scores in the practically defined “off” and “challenged-on” medication states were 38.5 ± 9.7 and 23.6 ± 12.0, respectively.

### Alpha/beta band power is attenuated during 130 Hz DBS but is not attenuated during 60 Hz DBS

**Fig 1:**
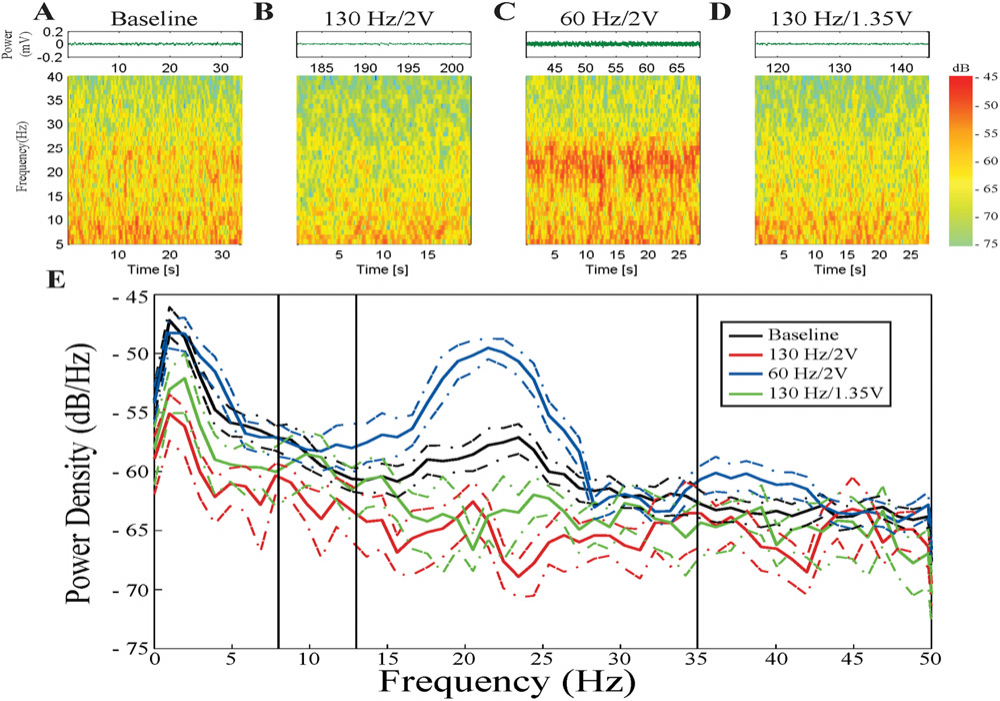
Example STN during the different stimulation sets. **A**, **B**, **C**, and **D** are raw waveforms and spectrograms from the baseline epoch, 130 Hz/2V DBS epoch, 60 Hz/2V DBS epoch, and 130 Hz/1.35V DBS epoch, respectively. Spectrograms are displayed with 99% window overlap. **E**: Relative PSD traces of the four epochs. Black = baseline; red = 130 Hz/2V; blue = 60 Hz/2V; green = 130 Hz/1.35V. Colored dashed lines represent 95% confidence intervals for each spectrum.

Fig. 1A-D displays the raw data and time frequency spectrograms from a representative STN, when the subject was in the resting state without DBS (Fig. 1A) and during different DBS epochs (Fig. 1B-D). Visual inspection demonstrated that baseline alpha/beta band spectral power was attenuated during 130 Hz DBS but amplified during 60 Hz/2V DBS.

The PSD in this example (Fig. 1E) confirmed this observation. In this subject during rest, there was increased LFP power at baseline in a sub-band (~17–26 Hz) within the beta band. During 130 Hz DBS at 1.35V, the LFP power in this frequency sub-band was attenuated, whereas during 130 Hz DBS at 2V, baseline LFP power was attenuated across a wider frequency range, from 8–35 Hz. In contrast, LFP power was not attenuated during 60 Hz/2V DBS. Instead, there was amplification of LFP power within the sub-band (~17–26 Hz). The effect of 60 Hz/2V DBS opposed that of the power-equivalent DBS set (130 Hz/1.35V).

The group analysis revealed significant attenuation of baseline LFP power in both the alpha and beta bands during 130 Hz/2V DBS (both P < 0.001) (Table 1). There was significantly greater attenuation of LFP power in the alpha and beta bands during 130 Hz/2V DBS compared to 130 Hz/1.35V DBS (alpha: P = 0.006; beta: P = 0.002). In contrast, during 60 Hz/2V DBS, there was significant amplification of baseline alpha band power (P = 0.012) (Table 1).

**Table 1:** Change (Δ) in Mean Power with Respect to Baseline.

*DBS Set (number of sides)*	*Alpha (8–12 Hz)*	*Beta (13–35 Hz)*
130 Hz/2V	Δ = -0.265	Δ = -0.431
(N = 14)	↓ P < 0.001	↓ P < 0.001
130 Hz/1.35V	Δ = -0.0685	Δ = -0.155
(N = 14)	↓ P = 1	↓ P = 0.193
60 Hz/2V	Δ = + 0.182	Δ = + 0.0932
(N = 14)	↑ P < 0.05	↑ P = 1

The total power delivered during 60 Hz/2V DBS was equivalent to that delivered during 130 Hz/1.35V DBS. However, their effects on baseline spectral power were significantly different from each other in both the alpha (P < 0.001) and beta (P = 0.006) bands.

### Patient-specific sub-bands of baseline LFP power are amplified during 60 Hz DBS

**Fig 2:**
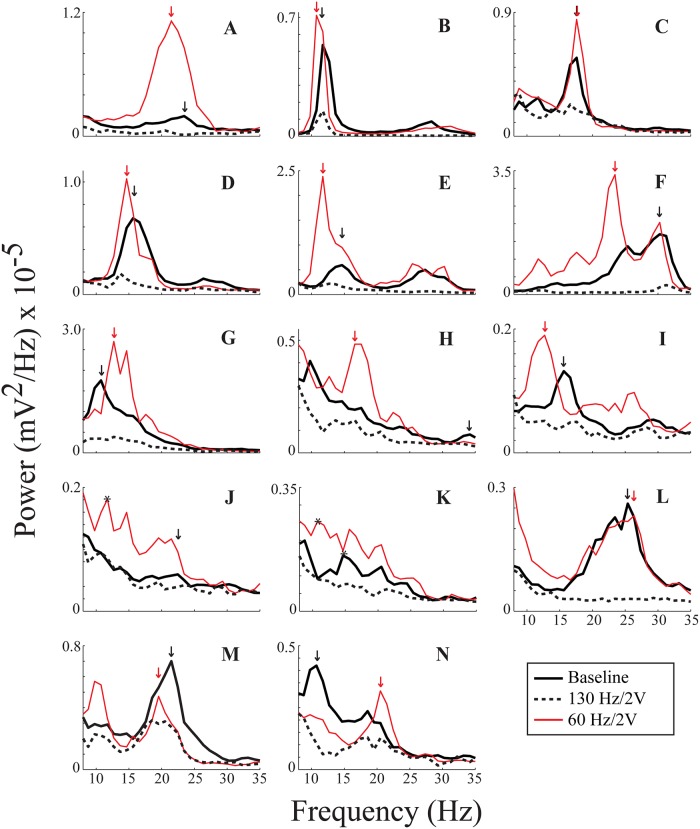
Peak detection for all fourteen sides. **A**—**N** are absolute PSDs from all fourteen recordings. Black = baseline; red = 60 Hz/2V; dashed = 130 Hz/2V. Arrows indicate where peaks were detected by the algorithm, and the color of an arrow corresponds to its PSD spectrum. Black asterisks on **J** (one for 60 Hz/2V) and **K** (two, one for 60 Hz/2V and one for baseline) indicate peaks chosen visually.

Fig. 2 demonstrates the resting state PSDs of LFP power from the DBS lead (across electrodes 0–2) from all fourteen sides at baseline, during 130 Hz/2V DBS, and during 60 Hz/2V DBS.

Baseline LFP power was attenuated in at least one sub-band of the alpha/beta band during 130 Hz/2V DBS in twelve out of the fourteen cases (Fig. 2). This was determined based on separation of the confidence intervals of the respective spectra, for which the upper confidence limit of the 130 Hz/2V DBS power spectrum was lower than the lower confidence limit of the baseline spectrum. In contrast, ten out of fourteen sides showed amplification of one or more sub-bands of the baseline alpha/beta band based on a similar analysis of separation of the lower confidence limit of the 60 Hz/2V DBS power spectrum and the upper confidence limit of the baseline spectrum. The effect of 60 Hz DBS varied among subjects: in three cases (Fig. 2F, J, and K), 60 Hz/2V DBS appeared to amplify certain sub-bands that were barely appreciated at baseline; and in three other cases (Fig. 2F, M, and N), similar sub-bands of LFP power were evident at baseline and during 60 Hz/2V DBS; however, the relative power switched between baseline and 60 Hz/2V DBS such that a different sub-band had the greatest power. In certain cases (Fig. 2F, I, and J), there appeared to be a downward shift of LFP power during 60 Hz/2V DBS that may have contributed to the significant amplification of LFP power within the alpha band demonstrated in the group band analyses (Table 1).

To quantify whether the peaks of the sub-bands of neural synchrony were greater during 60 Hz/2V DBS than at baseline, we focused on the peak with the maximum power in either state for each side. A conservative peak detection algorithm detected a peak during baseline and during 60 Hz/2V for twelve out of fourteen sides (arrows, Fig. 2). For the other two sides (Fig. 2J-K), maximum power peaks were chosen visually (black asterisks). Including these sides in the analysis, the maximum peak power was significantly greater during 60 Hz/2V DBS than at baseline (P = 0.007).

## Discussion

### Low frequency DBS increases STN neural synchrony

To our knowledge, this is the first study to investigate the effects of 60 Hz DBS on resting state neural synchrony in the STN in people with Parkinson’s disease and to compare such effects to that of high frequency (130 Hz) DBS in the same STNs.

During high frequency (130 Hz) DBS at 2 Volts, there was significant attenuation of both alpha and beta band LFP power, consistent with previous reports [7,10,18,34,35]. The effect of 130 Hz DBS at 1.35V was significantly different from that at 2V. We have previously shown that randomized presentations of different voltages of high frequency DBS produced a “dose” dependent attenuation of baseline resting state neural synchrony in the STN [18].

In contrast, lower frequency (60 Hz) DBS at 2 Volts did not attenuate STN alpha/beta band synchrony. In fact, there was a significant increase in alpha band (8–12 Hz) LFP power in the STN region during 60 Hz/2V DBS. The effect of 60 Hz DBS on STN neural synchrony did not appear to be due to lower total power delivered, as its effect opposed that of the power-equivalent DBS setting at 130 Hz (130 Hz/1.35V). This demonstrated that the effects of 60 Hz/2V DBS were most likely frequency-specific. These observed differences between 60 Hz DBS and 130 Hz DBS support the theory that these two frequencies have different effects on underlying neural circuitry.

The effect of intra-operative 60 Hz DBS on the resting state power spectrum profile was an amplification of one or more sub-bands of LFP power (neural synchrony), and the maximum peak power was greater during 60 Hz DBS than at baseline. In contrast, the effect of 130 Hz DBS at 2V was to flatten the spectra and eliminate the sub-bands of neural synchrony. There was significant variation among the resting state baseline spectral profiles as we have previously described, [28,32]. The effect of 60 Hz DBS also varied among cases; baseline sub-bands of neural synchrony were amplified in most cases during 60 Hz DBS and, in some cases, sub-bands that were not well appreciated at baseline became identifiable during 60 Hz DBS. In a few cases, the largest band of elevated LFP power switched between baseline and 60 Hz DBS. We do not believe that the heterogeneic effects of 60 Hz DBS on the STNs were due to different DBS lead locations; there was similarity among the effects on each STN within an individual (Fig. 2M-N) that were different from the effect on the two STNs of a different individual (Fig. 2A and L). We have previously shown that the baseline resting state LFP spectral peaks are similar and coherent between bilateral STNs of an individual but vary among PD subjects, and it was interesting that the effects of 60 Hz DBS also appeared to be patient-specific [28]. All the PD subjects have shown clinical improvement from STN DBS (data not shown), suggesting that the DBS lead was placed in or close to the sensorimotor region in the STN. The phenotype of PD subjects also varies so it is possible that this may contribute to different baseline LFP spectral profiles as well as the different effects of STN DBS. We have addressed this in a larger cohort of PD subjects but were not statistically powered to address this in the current group [32].

### Mechanisms of high frequency DBS

The STN is a critical node within the cortico-basal ganglia-thalamo-cortical network: it is interconnected with sensorimotor, associative, and limbic sub-/cortical structures [50–53]. This network consists of many nested loops whose oscillatory periods depend on the length of time it takes a signal to propagate around any given loop [54,55].

One of the proposed mechanisms of high frequency STN DBS is that the inter-pulse interval of the pulse train is similar to the period of certain nested loops within the network [21]. Thus, high frequency STN DBS may result in resonance within the sensorimotor network that overrides the pathological lower frequency synchrony in PD [21,22,24,56,57].

Another theory proposes that high frequency STN DBS directly reduces the imposition of pathological alpha/beta band synchrony on the STN by its action on the cortex. Pathological neural synchrony in the alpha/beta band in the sensorimotor region of the STN in PD may be locally derived, or it may be imposed from cortical regions either via cortico-striatal-pallidal-STN afferents due to the direct effect of dopamine depletion in the striatum or via the cortico-STN hyperdirect pathway (HDP) [18,19,58–62]. Experimental evidence using optogenetics demonstrated that activation of the cortico-STN HDP, either at its efferent projection site or via the HDP itself, replicated the behavioral effects of electrical STN DBS [63]. Recent theoretical models have predicted that STN DBS antidromic activation of the cortico-STN HDP would modulate cortical firing, leading to a disabling of excessive beta synchrony imposed by the cortex on the STN [64]. High frequency STN DBS thus may attenuate STN alpha/beta synchrony by antidromically inhibiting the cortical source(s) of these pathological rhythms.

### Proposed mechanism of sixty hertz DBS

It has been suggested that low frequency neurostimulation might have an inter-pulse interval that is too long to suppress intrinsic pathological and bursting activity within the STN. The stimulating spike train may combine with intrinsic pathological firing patterns to cause additional disruption of neural activity [21,24].

Our results suggest an alternative mechanism for 60 Hz DBS. We observed that in most cases, the effect of 60 Hz DBS was to amplify the already identified sub-bands of resting state neural synchrony, rather than to increase LFP power overall across the 8–35 Hz range. In some cases, sub-bands of neural synchrony that were not well appreciated at baseline became identifiable during 60 Hz DBS. We suggest that the mechanism of 60 Hz DBS is to invoke resonance in the patient-specific cortico-basal ganglia-thalamic-cortical loop(s) that are contributing to the baseline resting state neural synchrony in the STN and, in some cases, to invoke resonance in different cortico-subcortical loops that are not evident at baseline.

### Neurostimulation as an investigative tool for individuals and for individual behaviors in PD

Clinical evidence suggests that 60 Hz DBS may have a beneficial effect on axial motor behaviors, such as freezing of gait and speech, but not on tremor [36–41]. In contrast, high frequency DBS appears to be therapeutic for tremor but not always for freezing of gait or speech. However, results have varied among studies and among individuals. The results of this investigation demonstrated heterogeneity among STNs regarding the effect of 60 Hz DBS on baseline neural synchrony, and it is possible that this may contribute to the heterogenic clinical effects of 60 Hz DBS in PD reported so far. We have shown previously that the resting state STN spectral profile is stationary but varies among subjects [7,28,32]. We propose that one explanation for whether high or low frequency DBS is or is not therapeutic for different motor behaviors may lie in the resonant frequency of the nested loops pertinent to the networks mediating such behaviors. These may vary across the population, and may also be affected by amplitude- or frequency-modulation of interconnecting circuits. We demonstrate here that neurostimulation itself can be a powerful tool, whereby it is possible to “knock-in” and “knock-out” patient-specific bands of neural synchrony to determine their causal influences on human behaviors.

## Conclusion

This is the first study to investigate the effects of 60 Hz DBS on neural synchrony in the STN in people with Parkinson’s disease. Intra-operative 60 Hz DBS amplified baseline neural synchrony in contrast to the attenuation seen during high frequency (130 Hz) DBS. The effect on neural synchrony of 60 Hz/2V DBS opposed that of the total power-equivalent stimulation (130 Hz/1.35V DBS), suggesting that the effect is frequency- (not power-) dependent, and that high and low frequencies of DBS may resonate with different nested loops within the cortico-subcortical network. It may now be possible to selectively modulate individualized pathological “brain arrhythmias” in PD and other neuropsychiatric diseases to investigate their influence on specific motor and non-motor behaviors.
